# Study of Heavy Metal Accumulation and Residual Toxicity in Soil Saturated with Phosphate Processing Wastewater

**DOI:** 10.1007/s11270-017-3399-0

**Published:** 2017-05-25

**Authors:** Ali Mekki, Sami Sayadi

**Affiliations:** Laboratory of Environmental Bioprocesses, Center of Biotechnology of Sfax, AUF (PER-LBP), BP: 1177, 3018 Sfax, Tunisia

**Keywords:** Phosphate processing wastewater, Heavy metals, Soil, Microtoxicity, Phytotoxicity

## Abstract

The effects of phosphate processing wastewater (PPWW) on heavy metal accumulation in a Mediterranean soil (Tunisia, North Africa) were investigated. Moreover, the residual toxicities of PPWW-irrigated soils extracts were assessed. Results showed that heavy metal accumulation was significantly higher in PPWW-irrigated soil extracts than in control soil. The heavy metal accumulation increased over time in treated soil samples and their average values followed the following order: Iron (Fe 252.72 mg l^−1^) > Zinc (Zn 152.95 mg l^−1^) > Lead (Pb 128.35 mg l^−1^) > Copper (Cu 116.82 mg l^−1^) > Cadmium (Cd 58.03 mg l^−1^). The residual microtoxicity and phytotoxicity of the various treated soil samples extracts were evaluated by monitoring the bioluminescence inhibition (BI %) of *Vibrio ficheri* and the measurement of the germination indexes (GI %) of *Lepidium sativum* and *Medicago sativa* seeds. The results showed an important increase of residual toxicities of PPWW-treated soil extracts over time.

## Introduction

The phosphate industries are the cause of many environmental problems. Pope and Burnett (2002) confirms that the mining industry is a highly polluting activity. The mining industry is known by consuming space and producing wastes (Huerta et al. 2002). According to Jarvis and Burnett (1994), natural phosphates contain many metallic elements and therefore have negative impacts on the environment.

Mining, manufacturing, and the use of synthetic products can result in heavy metal contamination of urban and agricultural soils (Tu et al. 2000; Gnandi and Tchangbedji 2006; Nouri et al. 2008). The heavy metals are of special concern because they are non-degradable like carbon-based (organic) molecules and therefore persistent (Selene et al. 2003). These metals include Cd, Pb, Zn, Cu, nickel (Ni), mercury (Hg), and the metalloid arsenic (As) (Brigden et al. 2002; Gnandi and Tchangbedji 2006; Azumi and Bichi 2010; Thomas et al. 2012).

The heavy metals like Cd, Pb, and As have been found in P fertilizers and are considered the most important of health concern (Morgan 2013). These elements are regarded toxic and classified as carcinogenic (Farooqi et al. 2009). The exposure to acute Cd and Zn concentrations often results in gastrointestinal and respiratory damage, as well as damages to heart, brain, and kidney (Frieberg et al. 1992).

The Company of Phosphates of Gafsa (CPG) in Tunisia (North Africa) is one of the leading phosphate producer’s worldwide (Nasariah et al. 1988; Tijani and Fakhfakh 2011).

The phosphate processing is a transaction consisting of several phases, including mechanical preparation of separation and treatment to increase the P_2_O_5_ content (Nasariah et al. 1988). The separation of grains is fed by 5 m^3^ of water per ton of phosphates. However, from these five m^3^ of water, 3.65 m^3^ was recycled; 0.15 m^3^ accompanies the phosphate product as moisture and 1.2 m^3^ is abandoned (Nasariah et al. 1988). Thus, production of 8 million tons per year of marketable phosphate consumes approximately 10.5 × 10^6^ m^3^ of waters (Tijani and Fakhfakh 2011). Moreover, the waters used in phosphate processing were evacuated wildly in the nature which results harmful consequences on the receiving medium (Sharifi and Safari 2012).

The main objective of this study was to evaluate the heavy metal accumulation in a Mediterranean soil with arid climate (Tunisia, North of Africa) in responses to PPWW application and also to assess the residual microtoxicity and phytotoxicity of PPWW-irrigated soil extracts in function of incubation period.

## Materials and Methods

### PPWW Origin and Sampling

**Fig. 1:**
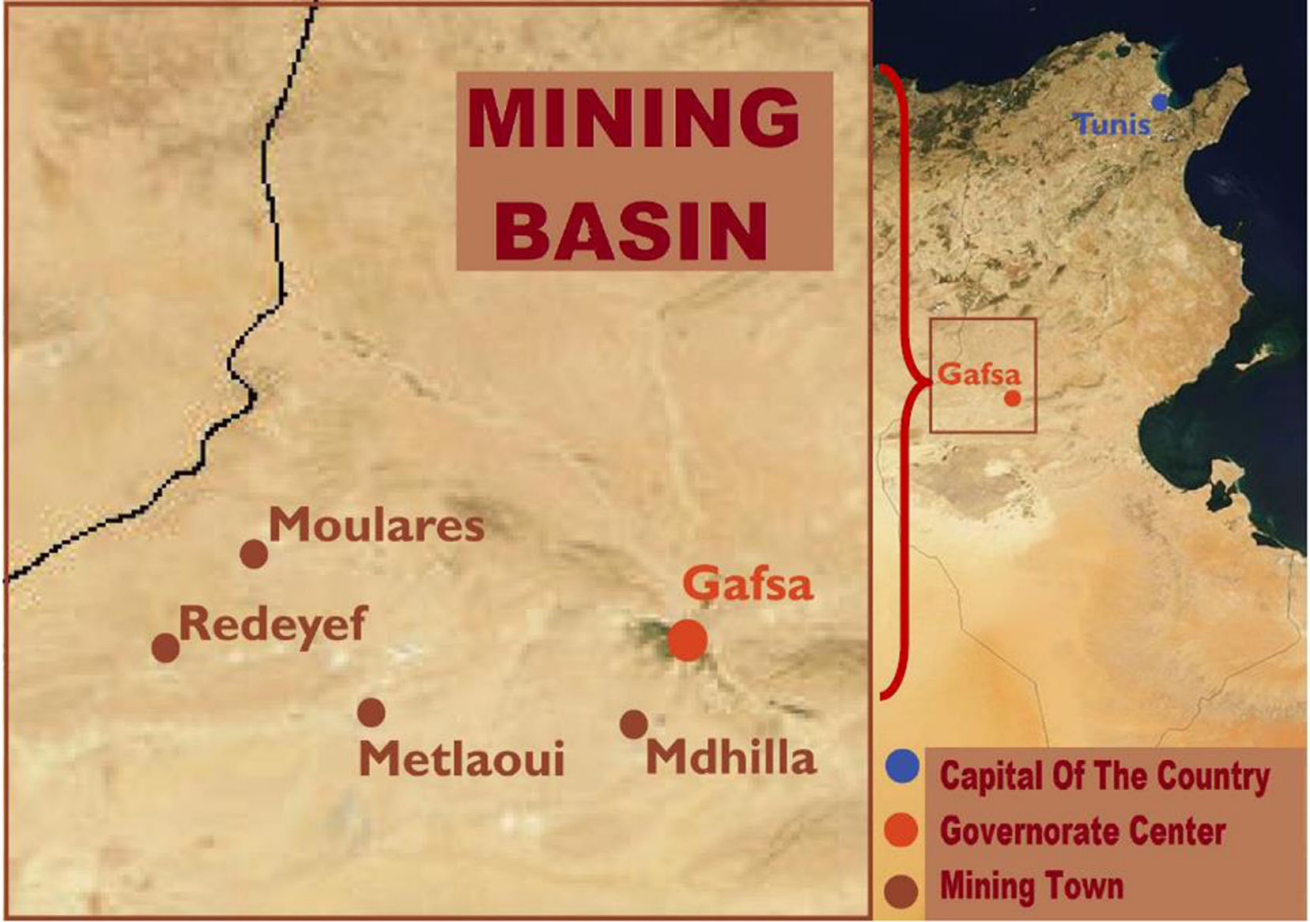
Geographical map of the Gafsa mining basin, Tunisia

The studied wastewater comes from the phosphate processing industry of the Company of Phosphates of Gafsa (CPG), at the South-West of Tunisia (Fig. 1). PPWW samples (15 samples, at the rate of 3 samples from each point) were taken at February 2016 from different points (5 points) of the exit of the laundries. The samples were kept at −4 °C until analyses.

### PPWW Physicochemical Analyses

pH measurement was carried out by a pH meter type STARTER 2100. The electrical conductivity (EC) was measured by a Conductimeter type Cond 1970i. The turbidity was measured by a turbidimeter type VTV.

The chemical oxygen demand (COD) was determined according to the Knechtel (1978) method. Five-day biochemical oxygen demand (BOD_5_) was determined by the manometric method with a respirometer.

Suspended matter, dry matter, organic matter, and mineral matter were determined according to Sierra et al. (2001). Total nitrogen, ammoniacal nitrogen, and organic nitrogen were determined according to Kandeler (1995) method.

Phosphorus (P), potassium (K), calcium (Ca) and heavy metals as Fe, Cd, Zn, Pb, and Cu were determined by atomic absorption spectrophotometer (AAS).

### Soil Description and Sampling

Soil samples were taken from uncultivated land located in the region of Sfax, South of Tunisia (North latitude 34°3′, East longitude 10°20′). Treated soil was saturated by PPWW at a rate of 50% of its holding capacity in comparison with the control soil (S_c_) irrigated with pure water. Treated and control soils were incubated in pots (6 pots as 3 pots for treated soils and 3 pots for control soil) for 120 days under ambient conditions. Soils samples (18 samples were taken, at the rate of 3 samples of each pot) were collected after 30 (S_30_), 60 (S_60_), 90 (S_90_), and 120 (S_120_) incubation days and at a depth of 0–20 cm. The moisture deficit during the incubation time was adjusted by adding pure water to maintain the soil microbiological activities.

### Soil Analyses

All PPWW-saturated and control soils samples were air dried, sieved at 2 mm, and analyzed for granulometric and physicochemical properties. Soil texture was determined using the pipette method (Thomas et al. 2012). The soil water-holding capacity was determined gravimetrically by saturating the soil overnight. The pH and electrical conductivity (EC) of soils were determined using a pH meter and an EC meter, respectively. Soil dry matters, organic matters, and mineral matters were determined according to Sierra et al.’s (2001) method. For the determination of soil total nitrogen and the ammoniacal nitrogen, the Kjeldahl method has been applied (Kandeler 1995).

Soil extract soluble elements (P, Ca, K, Na) and heavy metals (Cu; Zn; Fe; Cd; Pb) were determined by AAS.

### Assessment of PPWW Residuals Toxicities

#### Microtox Toxicity Test

The Microtox toxicity test uses the marine bioluminescent bacteria *Vibrio fisheri* as the test organism. The Microtox test has been used to measure the residual toxicity of all PPWW-irrigated soil sample extracts based on the bioluminescence inhibition (BI %) according to Aloui et al. (2007).

#### Phytotoxicity Tests

Residual phytotoxicity of all PPWW-irrigated soil sample extracts was evaluated by determination of seed germination index of *Lepidium sativum* and *Medicago sativa* seeds (Zucconi et al. 1981).

### Statistical Analyses

For each analyzed parameter, three repetitions were made for both the PPWW samples and the soil samples. Data were subjected to analysis of variance (ANOVA) using the statistical system (SPSS Inc., IL, USA, version 16.0). Mean values were compared using the least significant difference (LSD) test at *P* ≤ 0.05.

## Results and Discussion

### PPWW Physicochemical Characterization

#### pH and Electrical Conductivity

PPWW show neutral or slightly alkaline pH values. This alkalinity essentially lies in their high calcium and phosphorus contents (Table 1). Alternatively, PPWW pH meets the required standards (INNORPI “ground water discharge of waste water” 1989; APHA 2005). However, these effluents showed a high salinity (EC average values = 9.35 mS cm^−1^), which represents a serious risk of salinization of the receiving medium (Liu and Haynes Richard 2010).

**Table 1:** PPWW physicochemical characteristics in comparison with Tunisian standards (INNORPI “ground water discharge of waste water” 1989) and with American Public Health Association (APHA 2005) standards for wastewater discharge

Characteristics	PPWW (SD)	INNORPI (SD)	APHA (SD)
pH (25 °C)	7.42 (0.2)	8.5(0.2)	9 (0.2)
EC (mS cm^−1^) (25 °C)	9.35 (0.2)	7.00(0.2)	6 (0.2)
Turbidity (NTU)	605 (3)	70.00(2)	NI
COD (mg O_2_ l^−1^)	63.23 (3)	90.00(2)	70(2)
BOD_5_ (mg O_2_ l^−1^)	14.55 (0.5)	30.00(1)	≤ 30(1)
COD/BOD_5_	4.34 ± (0.1)	3.00(0.1)	NI
Suspended matter (g l^−1^)	19.16 (0.5)	0.03(0.01)	≤ 0.03(0.01)
Dry matter (g l^−1^)	29.46 (0.5)	NI	NI
Organic matter (g l^−1^)	4.46 (0.1)	NI	NI
Mineral matter (g l^−1^)	24.82 (0.5)	NI	NI
Total nitrogen (mg l^−1^)	43.42 (1)	90.00(2)	≤50(2)
Ammoniacal nitrogen (mg l^−1^)	31.73 (1)	NI	NI
Organic nitrogen (mg l^−1^)	11.66 (0.5)	NI	NI
P (mg l^−1^)	55.40 (2)	10.00(1)	≤10(1)
K (g l^−1^)	1.26 (0.02)	0.05 (0.01)	0.3 (0.01)
Ca (g l^−1^)	1.06 (0.02)	0.50 (0.01)	0.2 (0.01)
Na (g l^−1^)	0.85 (0.02)	0.50 (0.01)	0.2 (0.01)
Cu (mg l^−1^)	0.83 (0.02)	0.50 (0.01)	≤0.25 (0.01)
Zn (mg l^−1^)	0.94 (0.02)	5.00 (0.2)	≤1.00 (0.1)
Fe (mg l^−1^)	370.32 (3)	5.00 (0.2)	≤5 (0.2)
Cd (mg l^−1^)	1.21 (0.02)	0.1 (0.01)	≤0.1 (0.01)
Pb (mg l^−1^)	1.05 (0.02)	1.00 (0.01)	≤0.10 (0.01)

*PPWW* averaged values of three repetitions for each analysis, *SD* standard deviation (*P* ≤ 0.05), *NI* not identified

#### The Turbidity

Turbidity is the measure of relative clarity of a liquid. It refers to the content of a fluid material which makes it troublesome (Celik et al. 2010). In our case of study, the PPWW turbidity average values were about 605 UNT (Table 1). This value far exceeds the standards required for groundwater recharge with wastewaters (INNORPI “ground water discharge of waste water” 1989; APHA 2005). Indeed, turbidity depends on the quantity and the type of suspended particles present in the waste waters (Celik et al. 2010).

#### COD and BOD_5_

The COD average values of PPWW were around 63.23 mg l^−1^ while their BOD_5_ average values were about 14.55 mg l^−1^ (Table 1). These values were lower than standards required of wastewater discharges (INNORPI “ground water discharge of waste water” 1989; APHA 2005). However, the COD/BOD_5_ ratio that reflects biodegradability was well above the biodegradability values (Khoufi et al. 2006). This may be due mainly to the low value of BOD_5_, making difficult the biodegradation of such effluents. Alternatively, this leads to think that the use of plants in phytoremediation may be a potential treatment process for such type of effluents (Park et al. 2011).

#### Suspended Matter, Dry Matter, Organic Matter, and Mineral Matter

The determination of suspended matter (SM) is a very important parameter to assess the quality of effluent discharged and the appropriate treatment method (Rodier et al. 2005).

Table 1 shows the high levels of SM (19.16 g l^−1^) in PPWW. This level exceeds extremely the Tunisian standards required for the discharge of wastewaters (0.03 g l^−1^). Likewise and as shown in Table 1, most of the dry matter (DM) contained in the PPWW is in mineral form (MM = 24.82 g l^−1^) compared to the organic content present in these effluents (OM = 4.46 g l^−1^), confirming the preponderance of mineral matter in such effluents (Mignardi et al. 2013).

#### Nitrogen Content

The analysis of the nitrogen content of the PPWW shows that these effluents have an average total nitrogen Kjeldhal content around 43.42 mg l^−1^. This content was lower than the required standard (50 mg l^−1^) for discharge of wastewaters (INNORPI “ground water discharge of waste water” 1989; APHA 2005). Also, the mineral nitrogen content was more prominent than the organic nitrogen (Sou Dakouré 2013).

#### Phosphorus, Potassium, Calcium, and Sodium Contents

As shown in Table 1, the PPWW is very rich in potassium (1.26 g l^−1^), calcium (1.06 g l^−1^), and in sodium (0.85 g l^−1^) much more than phosphorus (55.40 mg l^−1^). This can be related to the phosphorus removal process inside the company (Tijani and Fakhfakh 2011). Indeed, the PPWW contents in potassium, calcium, sodium, and phosphorus exceed greatly the standards of discharges of wastewaters (INNORPI “ground water discharge of waste water” 1989; APHA 2005). Then, these elevated levels mainly in phosphorus presents a risk of soil contamination and waterway eutrophication (Sharifi and Safari 2012).

#### Heavy Metal Contents

PPWW heavy metal analyses were conducted for five metals such as Fe, Cd, Zn, Pb, and Cu. Results showed that the richness of PPWW in these metals was in the order Fe > Cd > Pb > Zn > Cu. Also, all PPWW metal contents exceed greatly the standards of discharges of wastewaters (Table 1). In line with this, several researchers have shown that phosphates are a major source of “heavy metals,” and some of which are radioactive (Bell et al. 1991). Indeed, the inorganic phosphorus used as fertilizer is often combined with toxic metals such as Cd, up to 87 mg kg^−1^ in a fertilizer product in Senegal (Jack 2010), and to radioactive elements, including uranium (U) of up to 390 mg kg^−1^ in Tanzanian mines against 12 mg kg^−1^ in the Tunisian mines of Gafsa (Nasariah et al. 1988).

### Effects of PPWW on Soil Granulometric and Physicochemical Characteristics

Soil is the top layer of the earth’s surface in which plants can grow, consisting of rock and mineral particles mixed with decayed organic matter.

Table 2 summarizes the granulometric and physicochemical characteristics of the soils studied. The texture of the control soil was predominantly sandy. The addition of PPWW for 120 days modifies this texture in favor of silts, which increases from 11.2% in the control soil to 17.7% in the soil irrigated with PPWW (Table 2). Thus, the textural class of soil moves from sandy to sandy loam, which increases the soil cation exchange capacity as well as heavy metal adsorption in the soil solid phase (Huerta et al. 2002).

**Table 2:** Granulometric and physicochemical PPWW-irrigated soil characteristics in comparison with control soil and in function of incubation time

Characteristics	Soil control (SD)	PPWW-irrigated soils			
		S30 (SD)	S60 (SD)	S90 (SD)	S120 (SD)
Sand (%)	76.45 (2) a	74.85 (2) a	73.55 (2) a	72.30 (2) a	69.40 (2)a
Clay (%)	12.35 (0.5)a	12.75 (0.5)a	12.85 (0.5)a	12.80 (0.5)a	12.9 (0.5)a
Silt (%)	11.2 (0.5)a	12.40(0.5)a	13.60(0.5)b	14.90(0.5)b	17.7(0.5)c
Textural class	Sandy	Sandy	Sandy	Sandy	Sandy loam
pH (25 °C)	7.80 (0.2)a	7.65 (0.2)a	7.70 (0.2)a	7.75 (0.2)a	7.70 (0.2)a
EC (mS cm^−1^)	0.53 (0.1)a	3.80 (0.2)b	3.98 (0.2)c	4.02 (0.2)c	4.05 (0.2)c
Dry matter (%)	94.15 (2)a	90.85 (2)a	92.75 (2)a	93.15 (2)a	93.35 (2)a
Water content (%)	05.85 (0.2)a	09.15 (0.2)b	07.25 (0.2)c	06.85 (0.2)d	06.65 (0.2)d
Organic matter (%)	01.23 (0.1)a	02.65 (0.1)b	02.45 (0.1)b	02.30 (0.1)b	02.15 (0.1)b
Mineral matter (%)	92.92 (2)a	88.20 (2)b	90.30 (2)b	90.85(2)b	91.20(2)b
Total nitrogen Kjeldhal (%)	0.05 (0.01)a	0.08 (0.01)b	0.09 (0.01)b	0.08 (0.01)b	0.07 (0.01)b
Ammoniacal nitrogen (%)	0.01(0.005)a	0.03 (0.005)b	0.04 (0.005)b	0.04(0.005)b	0.05(0.005)b
TOC (%)	0.71(0.05)a	1.53(0.05)b	1.41(0.05)b	1.32(0.05)b	1.24(0.05)b
C/N	14.2(0. 5)a	19.12(0. 5)b	15.66(0. 5)b	16.50(0. 5)b	17.41(0. 5)b
P (%)	0.02(0.005)a	0.21(0.01)b	0.19(0.01)b	0.22(0.01)b	0.23(0.01)b
Ca (%)	0.08(0.01)a	0.72(0.01)b	0.75(0.01)b	0.78(0.01)b	0.75(0.01)b
K (%)	1.00(0.01)a	2.65(0.02)b	2.70(0.02)b	2.85(0.02)b	2.75(0.02)b
Na (%)	0.06(0.01)a	0.23(0.01)b	0.25(0.01)b	0.35(0.01)b	0.37(0.01)b

Means followed within the same row by the same small letter are not statistically different

*SD* standard deviation (*P* ≤ 0.05)

The pH is considered to be the main chemical parameter controlling the bioavailability of heavy metals in soil (Brallier et al. 1996). The control soil pH was close to neutrality (7.8) while the average pH of the PPWW-irrigated soils was 7.7. In line with this, it has been demonstrated that the alkaline pH enhances heavy metal adsorption in the soil solid phase (Thornton 1996).

The mean values of the electrical conductivity (EC) in the different samples were variables. Thus, the control soil EC was in the order of 0.53 mS cm^−1^. The addition of PPWW to the soil considerably increases soil salinity. Indeed, the EC average value of PPWW-irrigated soils was about 3.9 mS cm^−1^. This is due to the initially high salinity of PPWW which was very rich in K, Ca, and Na. On the other hand, the salinity of soils irrigated with PPWW increases in function of the incubation time. This is consistent with findings confirming the increase in mineral content and salinity of wastewater-irrigated soils (Simmons 2009).

The analysis of the organic matter (OM) content in the various soil samples studied makes it possible to distinguish a relatively large increase in the control soil content compared to PPWW-irrigated soil (S_30_). However, this OM content decreases slightly in PPWW-irrigated soils with incubation time. This can be explained by the mineralization of the added organic matter (Madyiwa et al. 2002).

### Effects of PPWW on the Heavy Metal Accumulation in Soil

The role of heavy and trace elements in the soil system is increasingly becoming an issue of global concern at private as well as governmental levels (USDA 2001).

**Fig. 2:**
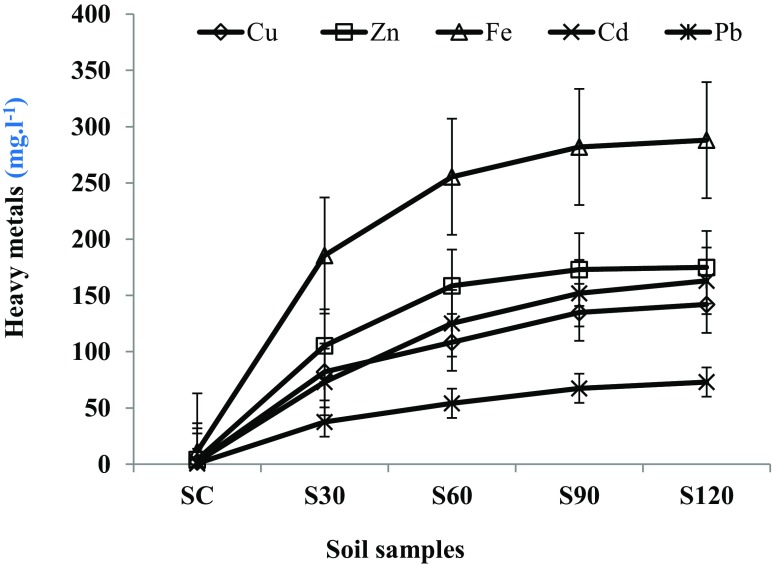
Heavy metal accumulation in PPWW-irrigated soil extracts in comparison with control soil extract and in function of incubation time

The heavy metal concentrations were evaluated in the PPWW-irrigated soil sample extracts in comparison of control soil sample extract. Mean total concentrations of five elements as Fe, Zn, Pb, Cu, and Cd were investigated. All the analyzed heavy metals showed to be higher in treated soil extracts than in control soil. Iron (Fe) concentrations were the most important and reach an average of 252 mg l^−1^ (Table 3), exceeding the maximal concentration of 100 mg l^−1^ admitted for ordinary soil (Bowen 1979). In line with this, Kisku et al. (2000) reported that among the different metals examined in soil, the concentration of Fe was higher than of other metals. Furthermore, our results showed the increase of various studied heavy metals in PPWW soil extracts over time incubation (Fig. 2). These results were in accordance with the findings of Thomas et al. (2012), who have demonstrated that the solubilization of heavy metals, increased with the time of soil saturation with wastewater. Also, many researchers showed that the solubilization of heavy metals differed among soils in the order sandy loam > loamy sand > sandy clay loam (Ahmed et al. 2009; Luo et al. 2012).

**Table 3:** Heavy metal levels in PPWW-irrigated soil extracts in comparison with control soil and ordinary soil (Bowen 1979) and in function of incubation time

Heavy metals	Soil control (SD)	PPWW-irrigated soil				Ordinary soil
		S30 (SD)	S60 (SD)	S90 (SD)	S120 (SD)	Bowen (1979)
Cu (mg l−1)	2.08(0.1)a	82.00 (2)b	108.30 (3)c	135.00(3)d	142.00 (3)d	30.00e
Zn (mg l−1)	4.15(0.5)a	105.35 (3)b	158.45 (4)c	173.00(5)d	175.00 (5)d	90.00b
Fe (mg l^−1^)	11.35(1)a	185.45 (5)b	255.16 (5)c	282.00 (5)d	288.00 (5)d	100.00e
Cd (mg l^−1^)	0.37(0.05)a	37.50 (2)b	54.12 (2)c	67.50 (2)d	73.00 (2)e	2.00f
Pb (mg l^−1^)	2.33(0.1)a	73.15(2)b	125.25 (3)c	152.00 (3)d	163.00 (3)e	35.00f

Means followed within the same row by the same small letter are not statistically different

*SD* standard deviation (*P* ≤ 0.05)

However, Islam et al. (2007) showed that some metallic elements are naturally present at traces levels in soils and some of these elements such as manganese (Mn), Zn, boron (Br), and Cu are essential or beneficial to allow normal development of organisms. Other elements such as Cd, mercury (Hg), and Pb are strict contaminants even in small quantities, and they accumulate in the soil because of their long biological half-life (Luo et al. 2003).

### Assessment of PPWW Soil Extract Residual Toxicity

#### Microtox Toxicity Test

The Microtox toxicity test is described as a potentially useful tool for the rapid toxicity testing of environmental samples including wastewater and soils irrigated (Aloui et al. 2007).

**Fig. 3:**
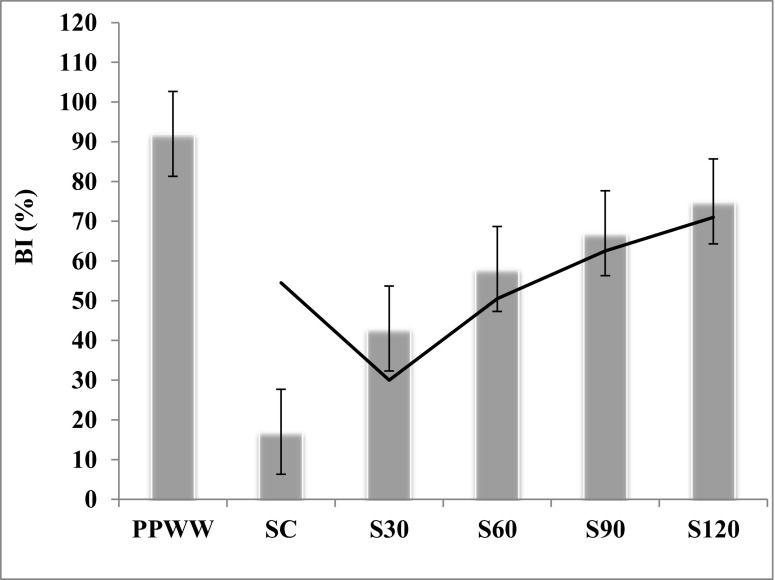
PPWW and irrigated soil extract microtoxicity in comparison with control soil extract and in function of incubation time

Our results show that this test is capable of detecting the toxicity of metals at parts-per-million (ppm, mg l^−1^) levels. PPWW was found to be the most toxic to *V. fisheri* with BI (%) around 92% (Fig. 3). The same results found that extracts of soils irrigated with PPWW showed very high residual toxicity levels in comparison with control soil extract. Moreover, these residual toxicities increase with the incubation time in soils (Fig. 3). In line with this, Madyiwa et al. (2002) showed that the level of the soil contamination with heavy metals depends on its physicochemical properties, concentration, and type of heavy metals in the water used and lastly how long the soil has been irrigated. Indeed, microbial toxicity tests rely on bacteria responding rapidly to changes in the environment and are easy and inexpensive to perform (Chi-Ying et al. 2004).

#### Seed Germination and Phytotoxicity Tests

Germination is a complicated physiological process of plant growth. The seedling test is generally used for evaluating phytotoxicity of waters or soils irrigated by extracted solutions (Zucconi et al. 1981).

**Fig. 4:**
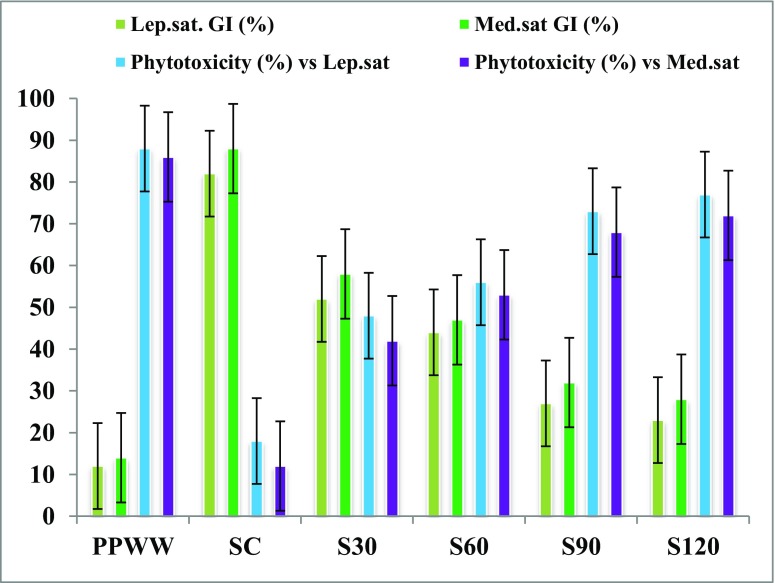
PPWW and irrigated soil extract phytotoxicity vs. *Lepidium sativum* (*Lep.sat.*) and on *Medicago sativa* (*Med.sat*) seeds in comparison with control soil extract and in function of incubation time

Effects of PPWW and soil irrigated extracts in comparison with control soil extract on the germination indexes (GI) of *L. sativum* and *M. sativa* seeds were investigated (Fig. 4).

Results showed that PPWW was very phytotoxic for both types of seeds studied. In fact, the GI (%) in the presence of PPWW was about 12% for *L. sativum* and about 14% for *M. sativa*, which results in phytotoxicity values of 88 and 86%, respectively, for *L. sativum* and *M. sativa*. PPWW-irrigated soil extracts show relatively higher GI (%) than PPWW does, and consequently, their residual phytotoxicities were lower compared to PPWW for the two types of seeds tested. However, the phytotoxic potential of PPWW-irrigated soils remains extremely high compared to the control soil extract. On the other hand, the results show that PPWW-irrigated soil extract phytotoxicities increase as a function of the incubation time (Fig. 4). These results were in agreement with the increase in the heavy metal content of the irrigated soils as a function of the incubation time (Table 3).

In line with this, Zucconi et al. (1985) reported that values for the GI lower than 50% mean high phytotoxicity, values between 50 and 80% mean moderate phytotoxicity, and values over 80% indicate that the material present is not phytotoxic. Based on these findings, in our study, PPWW, S_60_, S_30_, and S_90_ can be considered high phytotoxic for the two seeds tested, S_30_ can be considered as moderate phytotoxic, while control soil extract is not phytotoxic.

Indeed, previous reports clearly indicate a negative impact of heavy metals on seed germination (Athar and Ahmad 2002; Jamal et al. 2006). In fact, increasing concentrations of heavy metals in waters and soils irrigated gradually decrease germination percentage and thereby increase phytotoxicity (Isak et al. 2013).

## Conclusions

Our work has focused on the study of the impacts of PPWW application on the accumulation of heavy metals in soil and the assessment of their residual toxicities.

Our results showed the richness of PPWW on heavy metals. This richness was reflected in the accumulation of these elements in the soils irrigated. PPWW did not modify the control soil pH, but it increased its salinity. This is related to PPWW richness in salts and mainly in heavy metals. The heavy metal content detected in PPWW soils irrigated was too high in comparison with the permissible values for an ordinary soil.

Our results showed also that the content of heavy metals in soils increases with incubation time. This was accompanied by an increase in residual toxicities (microtoxicity and phytotoxicity). Indeed, these findings explain the direct correlation between the heavy metal contents and the residual toxicities of PPWW soil irrigated extracts.
